# Expression of the PXR gene in various types of cancer and drug resistance

**DOI:** 10.3892/ol.2013.1149

**Published:** 2013-01-22

**Authors:** ENQI QIAO, MINGHUA JI, JIANZHONG WU, RONG MA, XIAOHUA ZHANG, YUEJUN HE, QUANBIN ZHA, XUE SONG, LI-WEI ZHU, JINHAI TANG

**Affiliations:** 1 Department of General Surgery, Jiangsu Cancer Hospital, Affiliated to Nanjing Medical University, Nanjing 210009;; 2 Department of Oncology, The First Affiliated Hospital with Nanjing Medical University, Nanjing 210009;; 3 Department of General Surgery, Xuzhou Medical College, Xuzhou 221000, P.R. China

**Keywords:** pregnane X receptor, multidrug resistance, cancer, tumor cell apoptosis

## Abstract

Pregnane X receptor (PXR) is a member of the nuclear receptor superfamily of ligand-regulated transcription factors. PXR is a key xenobiotic receptor that regulates the expression of genes implicated in drug metabolism, detoxification and clearance, including drug metabolizing enzymes and transporters, suggesting that it is significant in the drug resistance of cancer cells. PXR is expressed in a wide range of tissues in the human body. Studies have demonstrated that PXR is expressed in a variety of tumor types, correlating not only with drug resistance but also with the cell proliferation, apoptosis and prognosis of cancer. The purpose of the present review is to provide a comprehensive review of PXR and its potential roles in multidrug resistance and the biological characteristics of PXR-positive tumors.

## Contents

1. Pregnane X receptor
2. Correlation between the expression of PXR in various types of cancer and drug resistance
3. PXR and tumor cell apoptosis
4. Preventing drug resistance by regulating PXR
5. Conclusions and prospects

## 1. Pregnane X receptor

### Nomenclature

Pregnane X receptor (PXR; NR1I2) belongs to the NR1I subfamily of nuclear receptors (NRs). PXR was discovered by several groups in 1998 (1–4) and is alternatively referred to as the steroid and xenobiotic receptor (PXR) and pregnane-activated receptor (PAR), also known as SXR or hPXR in humans.

### Structure of PXR

**Figure 1:**
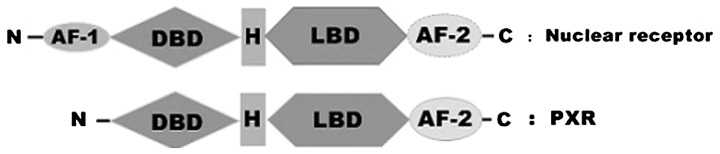
Schematic comparison of the domain structures of a typical nuclear receptor and PXR. AF-1, activation function-1; DBD, DNA-binding domain; H, hinge region; LBD, ligand-binding domain; AF-2, transactivation function-2; PXR, pregnane X receptor.

PXR contains the highly conserved DNA-binding domain (DBD), a characteristic structure of NRs. The far N-terminus is a short activation function-1 (AF-1) region which permits the regulation of receptor action in a ligand-independent manner. The structure of the PXR DBD is highly similar to that of the retinoid X receptor α (RXRa) DBD, which is a double zinc-finger motif that contacts DNA in a sequence-specific fashion. The response elements include direct repeats with 3 to 5 bases separating the DBD binding sites (DR-3, DR-4 and DR-5) and inverted repeats (with the beginning of each sequence in proximity) separated by 6 or 8 bases (ER-6 and ER-8, respectively) (1,4,5). The two most important PXR target genes, multidrug resistance protein 1 (MDR1) and cytochrome P450 3A4 (CYP3A4), contain DR-4/ER-6 (6) and DR-3/ER-6 (4,7) in their promoter regions, respectively. The PXR DBD is also reported to contain a bipartite nuclear localization sequence (8). The DBD is linked to the ligand-binding domain (LBD) in PXR by a hinge region which is considerably shorter than that observed in the majority of NRs. The LBD contains the ligand-dependent activation function 2 region and the ligand-binding pocket. It is possible for the LBD of PXR to heterodimerize with the LBD of RXRa, similar to the known structures of other NR LBDs with the RXRa LBDs (9). Conformational changes upon ligand binding in AF-2, which are responsible for the recruitment of coregulator proteins, lead to changes in the transcription of target genes (Fig. 1) (10–12).

**Figure 2:**
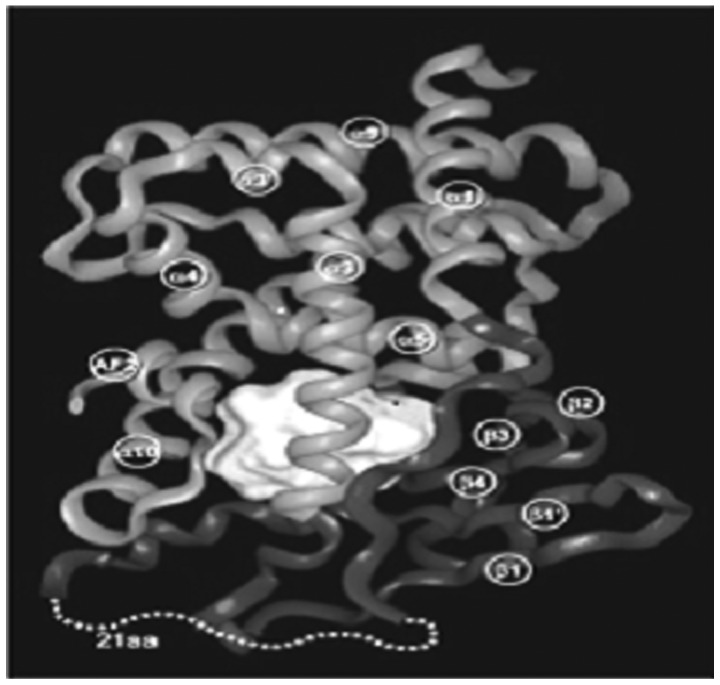
Structure of the PXR LBD. The three-dimensional structure of the human PXR LBD is presented as a ribbon diagram. The α-helices, β-sheets and the AF-2 helix are indicated. The helix 1-helix 3 insert, which is unique to PXR among the NRs, is indicated in dark grey. The large solvent-accessible ligand-binding pocket is outlined in white. PXR, pregnane X receptor; LBD, ligand-binding domain; AF-2, transactivation function-2; NR, nuclear receptor. This figure is taken from ref. 84.

The three-dimensional structure of the LBD in PXR reveals that it has a large, spherical ligand-binding cavity which enables it to interact with a wide range of hydrophobic chemicals. Structural flexibility appears to be extremely important to allow PXR to conform to a series of ligands that differ in size and shape. Therefore, unlike other NRs which interact selectively with their physiological ligands, PXR serves as a generalized sensor of hydrophobic substances (Fig. 2).

### Function of PXR

Unlike the majority of NRs, the list of genes which are regulated by PXR is growing rapidly, including not only the systems associated with drug and xenobiotic metabolism but also those central to cholesterol and bile acid metabolism and excretion.

PXR has been shown to regulate the expression of genes involved in the oxidation (phase I), conjugation (phase II) and transport (phase III) of xenobiotics, which promote the metabolism, elimination and detoxification of chemotherapeutic agents. Phase I drug metabolizing enzymes (DMEs), regulated by PXR, include a number of CYPs, carboxylesterases, alcohol and aldehyde dehydrogenases and enzymes involved in heme production and the P450 reaction cycle (13,14). PXR is a significant regulator of the xenobiotic-responsive expression of CYP3A genes. Accordingly, PXR is highly expressed in the human liver and intestine, where CYP3A is abundant and capable of metabolizing a broad range of structurally diverse xenobiotics (15,16). A large number of compounds that induce CYP3A expression are also PXR activators (17). Phase II DMEs which are regulated by PXR facilitate the excretion of phase I biotransformed xenobiotics, including glucuronyl transferases (UGTs), sulfotransferases (SULTs) and glutathione transfer-ases (GSTs) (18). UGT-catalyzed glucuronidation reactions are vital for the clearance of bilirubin and xenobiotics. A number of UGTs, including UGT1A1, UGT1A3, UGT1A4, UGT1A6 and UGT1A9, have been identified as PXR targets (19–24). Numerous efflux transporters, including ABC drug efflux transporters, breast cancer resistance protein (BCRP), multi-drug resistance-associated proteins (MRPs) and P-glycoprotein (P-gp) are also regulated by PXR (6,15,16,25,26).

PXR coordinately regulates a large proportion of genes and proteins in the liver, intestine and other organs that are involved in all aspects of the detoxification and elimination of xenobiotics and drugs.

PXR is unique in the NR superfamily since it responds promiscuously to a wide range of chemically-distinct ligands from 232 to >800 Da (1–3,25,27). The induction of CYP3A4 expression represents the basis for an important class of drug-drug interactions in which one drug accelerates the metabolism of other drugs. It is evident that the majority of the prescription drugs which induce the expression of CYP3A4 do so by activating PXR. Furthermore, PXR has also been associated with the interaction between the herbal remedy St. John’s wort (SJW) and prescription drugs (28). The knowledge that PXR is the molecular basis for common drug-drug interactions should contribute to the development of safer medicines.

Numerous studies have demonstrated new and mostly unexpected roles for PXR in regulating inflammation, bone homeostasis, energy homeostasis, lipid homeostasis and cancer.

### Distribution of PXR

The expression of PXR is widespread in the tissues of humans and rodents. PXR is highly expressed in the liver, small intestine and colon in humans, rabbits, rats and mice (1–4,27–30). Notably, these are also the same tissues where the *CYP3A* genes are most highly induced and expressed. In rodents, PXR mRNA has been detected in the kidney (31), brain (32), lung (33) stomach, ovary, placenta (34,35), immune cells (36), peripheral mononuclear blood cells (37,38), heart, bone marrow and spinal cord (39). PXR is expressed not only in normal tissues, but also in numerous types of human cancer, including breast (40,41), osteosarcoma (42), colon (43), endometrial (44,45), ovarian (46), prostate (47) and esophageal (48) cancers. Most significantly, the expression levels of PXR in these cancer tissues are usually higher than in non-neoplastic tissues.

## 2. Correlation between the expression of PXR in various types of cancer and drug resistance

### PXR and colon cancer

Of all types of cancer, studies of the correlation between PXR and colon cancer are the most common.

Pfrunder *et al*(49) noted that hPXR mRNA was highly expressed in three colon cancer cell lines, Caco-2 parental, Caco-2 TC-7 (TC-7) and LS180. Jiang *et al*(50) showed that the mRNA and protein levels of PXR and MRP3 were markedly higher in colon cancer tissues than in non-neoplastic tissues. MRP3 mRNA was significantly correlated with PXR mRNA in cancerous and non-neoplastic colon tissues. Furthermore, the protein level of MRP3 decreased following stable RNA interference against PXR. The authors also observed that PXR was able to enhance or reduce cell resistance to chemotherapeutic agents when activated by rifampicin (RIF) or knocked down via short hairpin RNAs (shRNA), respectively. The results suggest that PXR may be important in human colon cancer resistance to chemotherapeutics.

Harmsen *et al*(51) showed that tamoxifen, vincristine, vinblastine, flutamide, ifosfamide, docetaxel and paclitaxel were able to activate PXR-mediated P-gp induction and were also shown to affect the intracellular accumulation of the P-gp probe rhodamine 123. Moreover, PXR activation was also shown to reduce the cytotoxic activity of the P-gp substrate doxorubicin in colon cancer cells. The results indicated that several anticancer drugs are able to activate PXR-mediated induction of P-gp and affect the accumulation of P-gp substrates. Habano *et al*(52) demonstrated that PXR promoter methylation was involved in the regulation of intestinal PXR and CYP3A4 mRNA expression, which may be associated with the inter-individual variability in the drug responses of colon cancer cells.

Raynal *et al*(53) investigated whether PXR was markedly expressed in colon tumor samples and showed a great variability of expression. The expression of hPXR in human colorectal cancer cells led to a marked chemoresistance to SN38, the active metabolite of the anticancer drug irinotecan. This result demonstrated that PXR affected the tumoral metabolism of SN38 and suggested the potential therapeutic importance of PXR quantification in the prediction of the response to irinotecan. Basseville *et al*(54) reported that endogenous PXR is activated in response to SN38 in human colon cancer cell lines. The authors observed that endogenous PXR translocates into the nucleus and associates with RXR upon SN38 treatment. Using ChIP, the authors demonstrated that activated endogenous PXR binds to the native promoter of the CYP3A4 gene to induce expression. RNA interference experiments supported PXR’s involvement in CYP3A4 overexpression and allowed the identification of CYP3A5 and MRP2 transporter as PXR target genes. As a result, the authors observed that cells overexpressing PXR are less sensitive to irinotecan treatment. These results suggest that the PXR pathway is implicated in irinotecan resistance in a colon cancer cell line via the upregulation of specific detoxification genes.

Zheng *et al*(55) observed that the induction of CYP3A4 in human colon adenocarcinoma LS180 cells by hPXR agonist RIF led to a greater metabolic clearance of 1α,25-dihydroxyvitamin D (3) [1α, 25 (OH) (2)D (3)] and reduced the effects of the hormone on intestinal calcium absorption, which may contribute to an increased risk of drug-induced osteoporosis in patients receiving long-term therapy with hPXR agonists.

In addition, PXR has also been regarded as a regulator of the growth and apoptosis of colon tumors. Wang *et al*(56) used a xenograft model of colon cancer to define a molecular mechanism which may underlie PXR-driven colon tumor growth and malignancy. The activation of PXR was able to sufficiently enhance the neoplastic characteristics of human colon tumor cell lines and primary human colon cancer tissues xenografted into immunodeficient mice, including cell growth, invasion and metastasis. The authors also revealed that the PXR-mediated phenotype required FGF19 signaling. PXR bound to the FGF19 promoter in ‘normal’ intestinal crypt cells and human colon tumor cells. However, while the two cell types proliferated in response to PXR ligands, the FGF19 promoter was activated by PXR only in cancer cells. These data may lead to improved therapeutic regimens for colon cancer.

### PXR and breast cancer

Breast cancer is the most common cancer in women worldwide (57). Studies suggest that PXR has a potential clinically relevant role in breast cancer. However, the relevant pathway or target genes of PXR in breast cancer biology and progression have not yet been fully clarified. Dotzlaw *et al*(40) first detected the expression of PXR mRNA in normal and human breast tumor tissues. The expression of PXR mRNA did not differ between the tumor tissues and adjacent matched normal breast tissues, although the level of PXR mRNA did vary among the breast tumors. The authors also observed a statistically significant inverse correlation between the level of PXR mRNA and estrogen receptor (ER) status, but no correlation with progesterone receptor (PR) status. These data indicate the possibility that PXR has a potential role in human breast tissues. Conde *et al*(58) processed breast tissue samples from 99 patients, including *in situ*, infiltrative carcinomas and benign breast diseases, by immunohistochemistry and western blot analysis. The results showed that cancer cells from patients who developed recurrent tumors exhibited high cytoplasmic levels of hPXR isoform 1 and isoform 2, while infiltrative carcinomas that recurred showed a nuclear localization for hPXR and RXR-α. Therefore, the overexpression and the subcellular location changes of hPXR may be regarded as a potential new prognostic indicator.

Miki *et al*(41) detected PXR in carcinoma tissues but not in the non-neoplastic and stromal cells of breast cancers. A marked positive correlation was detected between the PXR labeling index and the histological grade and lymph node status of the carcinoma cases. PXR expression was also positively correlated with the expression of the cell proliferation marker Ki-67 in ER-positive cases. Microarray analysis showed that organic anion transporting polypeptide-A (OATP-A) was closely correlated with PXR gene expression and OATP-A mRNA and protein levels were significantly associated with PXR in breast carcinoma tissues and derived cell lines. Meyer zu Schwabedissen *et al*(59) also observed that the mRNA expression of OATP-1A2, a transporter capable of mediating the cellular uptake of estrogen metabolites, was ∼10-fold higher in breast cancer relative to adjacent healthy breast tissues. Of note, treatment of breast cancer cells *in vitro* with the PXR agonist RIF-induced OATP1A2 expression in a concentration-dependent and time-dependent manner. The RIF response was abrogated following small interfering RNA (siRNA) targeting of PXR. The authors used a novel potent and specific antagonist of PXR (A-792611) to show the reversal of the RIF effect on the cellular uptake of estrone 1-sulfate (E1S), an estrogen metabolite. The data indicate that PXR and its target gene may be key to the biology of human breast cancers and may also prove to be previously unrecognized targets for breast cancer treatment.

Sandanaraj *et al*(60) showed that PXR^*^1B was associated with reduced hepatic mRNA expression of PXR and its target genes, CYP3A4 and ABCB1. Genotype-phenotype correlations in breast cancer patients showed PXR^*^1B to be significantly associated with lower doxorubicin clearance, suggesting that PXR haplotype constitution may be important in influencing interindividual and interethnic variations in the effects of its putative drug substrates.

Choi *et al*(61) noted that TAM-resistant MCF-7 (TAMR-MCF-7) cells expressed higher levels of MRP2 than control MCF-7 cells. Molecular analyses using MRP2 gene promoters supported the involvement of PXR in MRP2 overexpression in TAMR-MCF-7 cells. Chen *et al*(62) also demonstrated hPXR expression in breast cancer cell lines and normal and cancerous human breast tissue specimens. Preactivation of hPXR by SR12813 in MCF-7 cells led to an increased resistance to tamoxifen. A significant increase in resistance to Taxol was also observed in MDA-MB-231 cells with hPXR preactivation. Following activation of hPXR, the expression of MDR1 and CYP3A4, two possible mediators of hPXR-mediated drug resistance in breast cancers was increased. Furthermore, a knock-down of hPXR through shRNA sensitized MCF-7 and MDA-MB-231 cells to treatment with tamoxifen, Taxol or vinblastine. Together, the data suggest a potential role for hPXR in breast cancer resistance to drug treatment.

### PXR and gynecological oncology

Masuyama *et al*(44) revealed various levels of PXR expression in endometrial cancer tissues but not normal tissues. Tissues showing high PXR expression exhibited markedly high expression of CYP3A4/7 and low expression of ER. HEC-1 cells, an endometrial cancer cell line, which express high PXR and low ER and PR, showed stronger transcriptional activity of the PXR-CYP3A pathway to the PXR ligands than Ishikawa cells. These results suggest that the steroid/xenobiotic metabolism may be important in the tumor tissue through PXR-CYP3A pathway, particularly in the alternative pathway for sex hormone and endocrine-disrupting chemical effects on endometrial cancer expressing low ER-α. The authors also examined whether endocrine-disrupting chemicals (EDCs) and anticancer agents were PXR ligands. PXR-mediated transcription was markedly activated by certain steroids/EDCs through the CYP3A4-responsive element compared with the MDR1-responsive element, whereas these steroids/EDCs also enhanced the expression of CYP3A4 compared with the expression of MDR1. However, anticancer agents, including cisplatin (CDDP) and paclitaxel, were able to markedly activate PXR-mediated transcription through the MDR1-responsive element compared with the CYP3A4-responsive element, whereas these drugs were also able to enhance the MDR1 expression compared with the CYP3A4 expression (63).

Gupta *et al*(46) studied the presence of PXR and its effects on ovarian cancer cells following activation by its cognate ligand. In SKOV-3 cells, an ovarian carcinoma cell line, the activation of PXR by cognate ligands induces target genes (CYP2B6, CYP3A4 and UGT1A1) but not MDR1 and MRP2. PXR activation also induced SKOV-3 cell proliferation and drug resistance. In mice with SKOV-3 xenografts, RIF, a PXR agonist, induced cancer cell proliferation and tumor growth. These data served as the basis for identifying novel inhibitors of PXR activation as an approach for controlling tumor growth and preventing induction of drug resistance.

Takami *et al*(64) used the CDDP-sensitive Ishikawa cell line and its CDDP-resistant sub-clone (ISIW^+^). ISIW^+^ cells showed higher PXR expression. When Ishikawa cells were cultured with PXR anti-sense oligonucleotides (AS), the cells did not gain CDDP resistance. In SCID mice, the authors observed that all AS-treated mice survived, whereas the controls had 50% survival at 35 days. The data indicated that PXR is a key factor for inducing, maintaining and reversing a CDDP-resistant phenotype in endometrial cancer cells. Yue *et al*(65) observed the expression of PXR and evaluated its clinical significance in human epithelial ovarian carcinoma. PXR was detected in 35 of 141 (24.8%) tumor tissues and showed significant differences with age, histology, grade, ER-α and PR. There was a statistically significant negative correlation between the PXR expression status and disease-free survival and overall survival. The results indicate an association of PXR with ER-α and PR in epithelial ovarian cancers. The data support PXR as a potential prognostic factor in epithelial ovarian cancer and PXR may serve as a useful marker for identifying patients at risk of recurrence or mortality.

### PXR and prostate cancer

Chen *et al*(47) first detected the expression of PXR in normal and cancerous prostate tissues. Pretreatment with SR12813 enhanced the resistance of PC-3 cells to Taxol and vinblastine. Futhermore, the PXR gene was knocked down, with PXR-targeting shRNA. The activity of PXR towards the promoter of CYP3A4 in PXR-ablated clones decreased compared with wild-type PC-3 cells. The cells’ sensitivities to Taxol and vinblastine were increased by PXR ablation. The data indicated that PXR may be important in prostate cancer resistance to chemotherapeutic agents.

Zhang *et al*(66) reported a novel PXR-mediated and metabolism-based mechanism for reducing androgenic tone. The study showed that genetic or pharmacological activation of PXR reduced the androgenic activity and inhibited androgen-dependent prostate regeneration in castrated male mice receiving daily injections of testosterone propionate by inducing the expression of hydroxysteroid SULT2A1 and CYP3As, which are enzymes significant for the metabolic deactivation of androgens. In human prostate cancer cells, treatment with the PXR agonist RIF inhibited androgen-dependent proliferation of LAPC-4 cells but had little effect on androgen-independent isogenic LA99 cells. Downregulation of PXR or SULT2A1 in LAPC-4 cells by siRNA abrogated the RIF effect, indicating that the inhibitory effect of RIF on androgens was PXR- and SULT2A1-dependent. In summary, PXR may be a potential therapeutic target for lowering androgen activity and may contribute to the treatment and prevention of hormone-dependent prostate cancer.

Fujimura *et al*(67) investigated PXR expression in human prostate tissues. The authors identified PXR immunore-activity using an anti-PXR antibody in benign (n=78) and cancerous (n=106) tissues obtained through radical prostatectomy. The authors analyzed the associations between the clinicopathological features of the patients, PXR status and CYP3A4 immunoreactivity. The experimental results showed differential PXR expression in human prostate tissues. High expression of PXR and CYP3A4 was a significant prognostic indicator of favorable outcomes in prostate cancer and may serve as a therapeutic target.

### PXR and liver cancer

Maruyama *et al*(68) showed that PXR mRNA was expressed in HepG2 cells, but not human fetal liver (HFL) cells.

To examine the role of PXR in hepatocellular carcinoma (HCC) as a receptor activated by vitamin K2, Azuma *et al*(69) established the cells stably overexpressing PXR using an HCC cell line, HuH7. Overexpression of PXR led to reduced proliferation and motility of the cells. More marked inhibition of cellular proliferation and motility was observed when PXR overexpressing clones were treated with vitamin K2. The data indicate that the activation of PXR may contribute to the tumor suppressing effects of vitamin K2 on HCC cells. The GADD45β gene is a direct target of PXR and stimulates cell signals to regulate various cellular functions. Kodama and Negishi (70) demonstrated that PXR activated the GADD45β gene, increased p38 MAPK phosphorylation and caused HepG2 cells to change morphology and migrate.

### PXR and esophageal cancer

Takeyama *et al*(48) performed immunohistochemical and quantitative RT-PCR evaluations in human esophageal squamous cell carcinoma (ESCC) in order to clarify the biological and clinical significance of PXR. The authors first immunolocalized PXR in 73 human ESCC cases. PXR immunoreactivity was detected in the cytoplasm and nuclei of carcinoma cells (20 and 98% of cases, respectively). The level of nuclear PXR immunoreactivity was inversely correlated with the histological grade, lymph node metastasis status, Ki-67/MIB1 labeling index and positively correlated with the RXR-α status. Furthermore, multivariate analysis further demonstrated that the PXR status in carcinoma cells was able to serve as an independent favorable prognostic indicator of the patients. The results of quantitative RT-PCR showed that PXR mRNA expression was detected in 60% of cases and was notably higher in the cancerous tissues compared with the non-neoplastic tissues of the patients. This was the first study to detect the status of PXR in human ESCC and the data indicate that PXR is a significant favorable prognostic factor of human ESCC.

van de Winkel *et al*(71) demonstrated PXR expression in Barrett’s esophagus (BE) and esophageal adenocarcinoma tissue and showed its nuclear localization in adenocarcinoma tissue. Upon activation with lithocholic acid, PXR translocated to the nuclei of OE19 adenocarcinoma cells. Together with the observed association of a PXR polymorphism and BE, the data suggest that PXR may have a potential role in the prediction and treatment of esophageal disease. The authors also revealed that PXR was able to separate high-grade dysplasia (HGD) from low-grade dysplasia (LGD) and no dysplasia (ND). PXR also appears to have diagnostic and prognostic value, but future prospective studies are required to investigate its predictive ability for neoplastic progression in BE (72).

### PXR and leukemia

Kawai *et al*(73) first identified the expression of PXR in HL-60 human promyelocytic leukemic cells in 2003.

All-trans-retinoic acid (ATRA) is an effective treatment for acute promyelocytic leukemia and several solid tumors, but its function is limited by resistance caused by increased metabolism. Wang *et al*(74) designed a study to demonstrate the role of PXR in ATRA metabolism. The study indicated that the coadministration of PXR ligands was able to increase the ATRA metabolism through the activation of the PXR-CYP3A pathway, which may be a mechanism for the ATRA resistance. Other PXR target transporters may also be implicated.

### PXR and osteosarcoma

Mensah-Osman *et al*(42) observed differences in the molecular size of the PXR protein expressed in sarcoma cell lines and the wild-type PXR expressed in the normal liver, small intestine or kidney. A polyclonal PXR antibody raised against the N-terminus of the wild-type PXR did not detect PXR expressed in the OS187, WOL and COL osteosarcoma cell lines. In these osteosarcoma cell lines, etoposide and doxorubicin were better inducers of P450 3A4 and MDR1 compared with RIF. siRNA against PXR down-regulated P450 3A4 expression levels only in the osteosarcoma cell line. Cytotoxicity assays indicated that the resistance of the osteosarcoma cell lines to etoposide correlated with the PXR protein expression levels and activation of P450 3A4 and was suppressed by ketoconazole. The results suggest that PXR is important in the regulation of P450 3A4 expression in osteosarcoma and its expression and activation may influence the effects of chemotherapeutic agents which induce PXR target genes implicated in drug resistance.

PXR expression is low or nonexistent in the lung, stomach, pancreas, kidneys, brain and other organs and there have been few studies of the correlation between PXR and these organic tumors at present.

## 3. PXR and tumor cell apoptosis

Studies have demonstrated that PXR is implicated in the apoptosis of tumor cells and may be an important factor in MDR.

In 2005, Zucchini *et al*(75) demonstrated that PXR expression was required for Bcl-2 and Bcl-xL upregulation upon PXR activator treatment in human and rat hepatocytes. PXR may protect the liver against harmful chemicals by simultaneously regulating detoxification and the cell apoptotic pathway.

Wang *et al*(76) examined the role of PXR in lipopolysaccharide (LPS)/D-galactosamine (GalN)-induced acute liver injury using PXR-null and wild-type mice. LPS/GalN-treated PXR-null mice had more marked increases in alanine transaminase (ALT), hepatocyte apoptosis, necrosis and hemorrhagic liver injury compared with wild-type mice. LPS/GalN-mediated phosphorylation of JNK1/2 and ERK1/2 was differentially regulated in PXR-null and wild-type mice. In addition, LPS/GalN-induced hepatic Stat3 survival signaling was impaired and early activation of Jak2 was delayed in PXR-null mice. After LPS/GalN treatment, the expression levels of the pro-survival proteins heme oxygenase-1 (HO-1) and Bcl-xL, which are downstream of Stat3, were markedly lower in PXR-null compared with wild-type mouse livers. The lack of PXR resulted in a significant reduction of LC3B-I and -II as well as Beclin-1 protein levels following LPS/GalN treatment. PXR is also implicated in hepatocyte homeostasis. Taken together, the results indicate that PXR is a key hepato-protective factor.

Masuyama *et al*(45) demonstrated that PXR overexpression led to a marked decrease in endometrial cancer cell growth inhibition and inhibited apoptosis in the presence of CDDP or paclitaxel. The data implied that PXR downregulation may be a novel therapeutic approach for the augmentation of sensitivity to anticancer agents or even to overcome resistance to them, in the treatment of endometrial cancer.

In a previous study, SuperArray analysis showed that PXR-mediated deoxycholic acid resistance was associated with the upregulation of multiple anti-apoptotic genes, including BIRC2, BAG3 and MCL-1 and downregulation of proapoptotic genes, such as TP53/p53 and BAK1 in human colon cancer cells (43).

However, a number of studies produced the opposite results. Ouyang *et al*(77) showed that PXR suppressed the proliferation and tumorigenicity of colon cancer cells by controlling the cell cycle at the G_0_/G_1_ phase by regulating the E2F/Rb and p21 (WAF1/CIP1) pathways. Verma *et al*(78) also observed that the activation of PXR was antiproliferative in p53 wild-type breast cancer cells and this effect was mechanistically dependent upon the local production of NO and NO-dependent upregulation of p53. This finding revealed a novel biological function of PXR and suggested that a subset of PXR activators may serve as effective therapeutic and chemo-preventative agents for certain types of breast cancers. Liu *et al*(79) also investigated whether Tanshinone IIA (Tan IIA) has significant growth inhibition effects on U-937 cells (a human leukemic monocyte lymphoma cell line) through the induction of apoptosis. Tan IIA-induced apoptosis may result from the activation of PXR, which inhibits the activity of NF-κB and leads to the downregulation of monocyte chemoattractant protein (MCP)-1 (MCP-1/CCL2) expression.

## 4. Preventing drug resistance by regulating PXR

The hypothesis that downregulating PXR in PXR-positive cancers increases the sensitivity of cancer cells to chemotherapeutic agents has been proposed and investigated in several studies. As previously mentioned, Masuyama *et al*(44) showed that the downregulation of PXR by siRNA in the endometrial cancer cell line HEC-1 decreased the expression of MDR1 and sensitized cells to the anticancer agents paclitaxel and CDDP. Chen *et al*(61) showed that treatment with the PXR agonist SR12813 activated PXR in breast cancer cell lines and increased the expression of MDR1 and CYP3A4 and the resistance of cells to paclitaxel, vinblastine and tamoxifen. By contrast, the targeted knockdown of PXR using shRNA enhanced the sensitivity of the cells to the anticancer drugs.

One approach to overcoming PXR activation is to chemically modify the lead compound and remove its PXR-activating function without compromising the target activity. Several studies have shown this concept to be possible in principle. For example, docetaxel and paclitaxel, two inhibitors of microtubule disassembly, have minor structural differences and equal potencies in inhibiting microtubule depolymerization and cancer cell proliferation. However, paclitaxel, but not docetaxel, significantly activates PXR and regulates MDR1 expression (25). Zimmermann *et al*(80) reported that chemical modifications to the first generation IGF-1R inhibitors reduce PXR transactivation while maintaining potency against IGF-1R. However, as a result of the agonist promiscuity of PXR, an extremely large amount of effort is required in drug development programs to remove the PXR activity while maintaining the target activity for the lead compounds.

PXR antagonists that cause PXR inhibition have been demonstrated to competitively bind to PXR using *in vitro* binding assays. Ecteinascidin-743 (ET-743), an antineoplastic agent, has been demonstrated to suppress PXR transactivation (25). A-792611, a HIV protease inhibitor, inhibits PXR-mediated CYP3A4 expression (81). Ketoconazole, an inhibitor of CYP3A4 enzyme activity, is able to inhibit a number of NRs, including PXR, by disrupting the NR-coactivator interaction (82). Sulforaphane (SFN), an inhibitor of histone deacetylases and an inducer of phase II DMEs, shows PXR antagonist activity (83). SFN down-regulates CYP3A4 expression by directly binding to PXR and inhibiting coactivator recruitment. Raynal *et al* showed that the activation of PXR reduced the chemosensitivity of colorectal cancer cells to irinotecan. Notably, the reduction in chemosensitivity could be reversed by SFN (53).

## 5. Conclusions and prospects

Overall, the expression of PXR is high in numerous types of tumors. Studies have demonstrated that PXR has a significant role in the drug resistance, proliferation, apoptosis and invasion of PXR-positive tumor cells. Therefore, PXR may be treated as a potentially key target in comprehensive cancer treatment and the prevention of drug resistance by regulating PXR expression is a novel and effective approach for oncotherapy.

PXR was discovered relatively recently and its structure and function have not yet been fully clarified. The correlation between PXR and multidrug resistance of tumors requires further study.
